# Association of intraoperative hypotension and cumulative norepinephrine dose with postoperative acute kidney injury in patients having noncardiac surgery: a retrospective cohort analysis

**DOI:** 10.1016/j.bja.2024.11.005

**Published:** 2024-12-12

**Authors:** Bernd Saugel, Michael Sander, Christian Katzer, Christian Hahn, Christian Koch, Dominik Leicht, Melanie Markmann, Emmanuel Schneck, Moritz Flick, Karim Kouz, Kerstin Rubarth, Felix Balzer, Marit Habicher

**Affiliations:** 1 Department of Anesthesiology, Center of Anesthesiology and Intensive Care Medicine, University Medical Center Hamburg-Eppendorf, Hamburg, Germany; 2 Outcomes Research Consortium, Cleveland, OH, USA; 3 Department of Anaesthesiology, Intensive Care Medicine and Pain Medicine, University Hospital Giessen, Justus-Liebig University Giessen, Giessen, Germany; 4 Institute of Medical Informatics, Charité - Universitätsmedizin Berlin, Corporate Member of Freie Universität Berlin and Humboldt-Universität zu Berlin, Berlin, Germany; 5 Institute of Biometry and Clinical Epidemiology, Charité - Universitätsmedizin Berlin, Corporate Member of Freie Universität Berlin and Humboldt-Universität zu Berlin, Berlin, Germany

**Keywords:** acute kidney injury, anaesthesia, blood pressure, cardiovascular dynamics, haemodynamic monitoring, hypotension, vasopressor

## Abstract

**Background:**

Intraoperative hypotension is associated with acute kidney injury (AKI). Clinicians thus frequently use vasopressors, such as norepinephrine, to maintain blood pressure. However, vasopressors themselves might promote AKI. We sought to determine whether both intraoperative hypotension and cumulative intraoperative norepinephrine dose are independently associated with postoperative AKI in patients undergoing noncardiac surgery.

**Methods:**

This was a retrospective cohort analysis of 38 338 adult male and female patients who had noncardiac surgery. The primary outcome was AKI within the first 7 postoperative days. We performed adjusted multivariable logistic regression analysis to determine whether intraoperative hypotension (quantified as area under a mean arterial pressure [MAP] of 65 mm Hg) and cumulative intraoperative norepinephrine dose were independently associated with AKI.

**Results:**

The median (25th percentile, 75th percentile) area under a MAP of 65 mm Hg was 0.09 (0.02, 0.22) mm Hg∗day in patients with AKI and 0.05 (0.01, 0.14) mm Hg∗day in patients without AKI (*P*<0.001). The cumulative intraoperative norepinephrine dose was 1.92 (0.00, 13.09) μg kg^−1^ in patients with AKI and 0.00 (0.00, 0.00) μg kg^−1^ in patients without AKI (*P*<0.001). Both the area under a MAP of 65 mm Hg (odds ratio 1.55 [95% confidence interval 1.17–2.02] per mm Hg∗day; *P*=0.002) and the cumulative intraoperative norepinephrine dose (odds ratio 1.02 [95% confidence interval 1.01–1.02] per μg kg^−1^; *P*<0.001) were independently associated with AKI.

**Conclusions:**

Both intraoperative hypotension and cumulative intraoperative norepinephrine dose were independently associated with postoperative AKI in patients undergoing noncardiac surgery. Pending results of trials testing whether these relationships are causal, it seems prudent to avoid both profound hypotension and high norepinephrine doses in adults undergoing noncardiac surgery.

**Editor's key points**

• Intraoperative hypotension is common and is associated with postoperative acute kidney injury (AKI) in noncardiac surgery patients. Therefore, treatment with vasopressors is routine. However, it is unclear whether the treatment is worse than the disease, as vasopressors are also associated with AKI.
• This single-centre retrospective cohort study analysed perioperative data from 38 338 adult patients having noncardiac surgery.
• In multivariable models that included a range of potential confounding factors, intraoperative hypotension (mean arterial pressure [MAP] <65 mm Hg), and intraoperative norepinephrine dose were independently associated with AKI.
• These data leave anaesthesia providers in an interesting position. As the study was observational, a causal relationship has not been established. It is not clear when, and if, randomised trials will confirm the respective impacts of intraoperative hypotension and intraoperative vasopressors on AKI.
• In the interim, anaesthesia providers should aim to avoid intraoperative hypotension and the need for treatment, as well as limit the duration of hypotension and the dose of vasopressors used for treatment.

Intraoperative hypotension is common in patients having noncardiac surgery with general anaesthesia,^1^^,^^2^ and is associated with organ injury, including acute kidney injury (AKI).^3^, ^4^, ^5^, ^6^ On a population basis, the hypotensive harm threshold for AKI is a mean arterial pressure (MAP) of around 65 mm Hg.^7^ Maintaining MAP above this level during surgery is therefore recommended.^8^^,^^9^

To maintain MAP and avoid intraoperative hypotension, clinicians frequently use vasopressors such as norepinephrine.^10^ While vasopressors are effective against hypotension, they themselves are associated with AKI.^10^, ^11^, ^12^ Norepinephrine, for example, may reduce renal perfusion by preferentially binding to α-receptors on renal afferent arterioles, resulting in renal medullary hypoxia.^13^

We assumed that not only intraoperative hypotension but also norepinephrine administered during surgery may be independently associated with postoperative AKI. We thus primarily sought to determine whether both intraoperative hypotension and the intraoperative cumulative norepinephrine dose are independently associated with postoperative AKI in male and female adults having noncardiac surgery. A previous analysis reported that intraoperative hypotension was associated with postoperative AKI in high-risk patients but not in low-risk patients.^14^ As a secondary analysis, we therefore repeated our primary analysis separately for patients with low *vs* high baseline risk according to the American Society of Anesthesiologists (ASA) physical status.

## Methods

### Study design, patients, and data sources

This was a retrospective cohort analysis of patients who had noncardiac surgery with general anaesthesia at the University Hospital Giessen (UKGM Giessen GmbH, Giessen, Germany) affiliated with the Justus-Liebig University Giessen between September 1, 2010 and September 7, 2020. The ethics committee of the Justus-Liebig University Giessen initially approved this study on October 10, 2019 (Az 194/19) and approved an amendment to extend the study period until September 7, 2020 on May 18, 2021. The sampling frame was based on the availability of suitable data. The need for informed consent was waived. The statistical analysis plan was written and approved by the principal investigators and study statisticians before data analysis.

We included male and female adults who had noncardiac surgery with general anaesthesia lasting >60 min (from the beginning of general anaesthesia until the end of surgery). We excluded patients with chronic kidney disease stage 5 (preoperative estimated glomerular filtration rate <15 ml min^−1^ (1.73m)^−2^), body mass index >100 kg m^−2^, missing preoperative baseline serum creatinine values within 30 days of surgery, need for renal replacement therapy, history of kidney transplantation, less than 10 MAP values per surgery, or MAP values min^−1^ <0.3. Patients who had major surgery on the genitourinary system (e.g. nephrectomy) and patients assigned ASA physical status 6 were also excluded. In patients who had repeated surgeries during the study period, only the first surgery was considered. Data were extracted from the patient data management system (ICUData) and the anaesthesia information management system (NarkoData) (both Imeso-IT GmbH, Giessen, Germany).

### Clinical management

Patients were monitored and managed per institutional routine, which included that clinicians generally strove to keep MAP >60–70 mm Hg. Vasopressors routinely used to treat hypotension in our institution are a 20:1 mixture of cafedrine/theodrenaline (approved in Germany as Akrinor®; Ratiopharm, Ulm, Germany) or norepinephrine (Arterenol®; Sanofi-Aventis Deutschland GmbH, Frankfurt am Main, Germany). Cafedrine/theodrenaline mediates its effects by stimulation of α- and β-receptors and nonspecific inhibition of phosphodiesterases, thereby increasing MAP by combined effects on preload, contractility, and afterload.^15^^,^^16^ Cafedrine/theodrenaline is used to treat intraoperative hypotension.^17^ It is administered as boluses and has long-lasting haemodynamic effects.^18^ Norepinephrine also can be administered as a bolus, but is usually given as continuous infusion because of its short-lasting haemodynamic effects. We routinely give norepinephrine via peripheral venous access.^19^^,^^20^

Blood pressure was measured either intermittently using oscillometry at 3-min intervals or continuously using an arterial catheter, and was recorded by the anaesthesia information management system at 3-min (oscillometry) or 1-min (arterial catheter) intervals.

### Hypotension exposure

We defined intraoperative hypotension as a MAP <65 mm Hg.^7^ Considering all blood pressure measurements recorded between the beginning of general anaesthesia and the end of surgery, we calculated the cumulative duration patients had a MAP <65 mm Hg (unit: min) and the area under a MAP of 65 mm Hg (unit: mm Hg∗day).^21^

### Norepinephrine exposure

We calculated the cumulative intraoperative norepinephrine dose, that is, the cumulative amount of norepinephrine administered to patients between the beginning of general anaesthesia and the end of surgery, considering both bolus and continuous administration. We normalised the cumulative norepinephrine dose to body weight (unit: μg kg^−1^).

### Outcomes

The primary outcome was AKI within the first 7 postoperative days. Postoperative AKI was defined based on the Kidney Disease: Improving Global Outcomes (KDIGO) Clinical Practice Guideline for Acute Kidney Injury^22^ as (a) an increase in serum creatinine concentration of ≥0.3 mg dl^−1^ within any 48 h period within 7 postoperative days, or (b) an increase in serum creatinine of ≥50% from baseline within the first 7 postoperative days, or (c) need for renal replacement therapy within the first 7 postoperative days. We did not consider urine output criteria as urine output was not systematically assessed and documented in our patients. The preoperative baseline serum creatinine concentration was defined as the most recent recorded value within 30 days before surgery. We considered AKI as a binary outcome (no AKI *vs* AKI of any stage).

### Statistical analysis

Variables are described using absolute numbers (percentages) for categorical variables, means (standard deviations) for normally distributed metric variables, and medians (25th percentile, 75th percentile) or ranges for non-normally distributed metric variables. Metric variables were assumed to be non-normally distributed if the kurtosis of the distribution exceeded 5.

We computed bar charts to illustrate the incidence of AKI stratified by both quartiles of intraoperative cumulative norepinephrine dose and quartiles of intraoperative hypotension in all patients irrespective of their ASA physical status, and separately in patients assigned ASA physical status 1 or 2 *vs* 3 and 4 (the few patients assigned ASA physical status 5 were analysed together with those assigned ASA physical status 4).

We compared patients with and without AKI within the first 7 postoperative days using two-sided *t*-tests for normally distributed variables, two-sided Wilcoxon-rank sum tests for non-normally distributed variables, or Χ^2^ tests for categorical outcomes. We performed univariable and multivariable logistic regression analyses to determine whether the area under a MAP of 65 mm Hg and the cumulative intraoperative norepinephrine dose were associated with AKI within the first 7 postoperative days. We adjusted multivariable logistic regression analysis for potentially confounding and mediating factors, which were selected by clinical rationale. Additionally, we constructed two directed acyclic graphs to conceptualise the relationships between the exposures (area under a MAP of 65 mm Hg and cumulative intraoperative norepinephrine dose) and the outcome (AKI within the first 7 postoperative days), while accounting for potential confounding and mediating variables (Supplementary Figs S1 and S2). The selection of confounders and mediators in the two directed acyclic graphs resulted in an identical adjustment set for both exposure–outcome models. Therefore, we could estimate the direct effect of the area under a MAP of 65 mm Hg and the total effect of cumulative intraoperative norepinephrine dose on AKI within the first 7 postoperative days in one model. However, we acknowledge the presence of unmeasured confounding factors; our analyses thus cannot confirm causal relationships and should be considered descriptive and exploratory.

We primarily performed multivariable logistic regression analysis including all patients irrespective of their ASA physical status. To investigate whether ASA physical status is also an effect modifier, we separately performed two multivariable logistic regression analyses: one including only patients assigned ASA physical status 1 or 2, and one including only patients assigned ASA physical status 3 and 4 (the few patients assigned ASA physical status 5 were analysed together with those assigned ASA physical status 4).

The significance level was set to *P*<0.05, and no adjustment for multiplicity was applied because of the exploratory nature of the study. Hence, we focused on effect sizes (i.e. odds ratios with 95% confidence intervals [95% CI]) and interpreted them in an exploratory manner.

We performed statistical analyses in R, Version 4.2.1 (R Foundation for Statistical Computing, Vienna, Austria) and used the following packages: MASS, readxl, plyr, magrittr, knitr, haven, mosaic, kableExtra, grid, gridExtra, tidyverse, tableone, broom.

## Results

**Fig 1:**
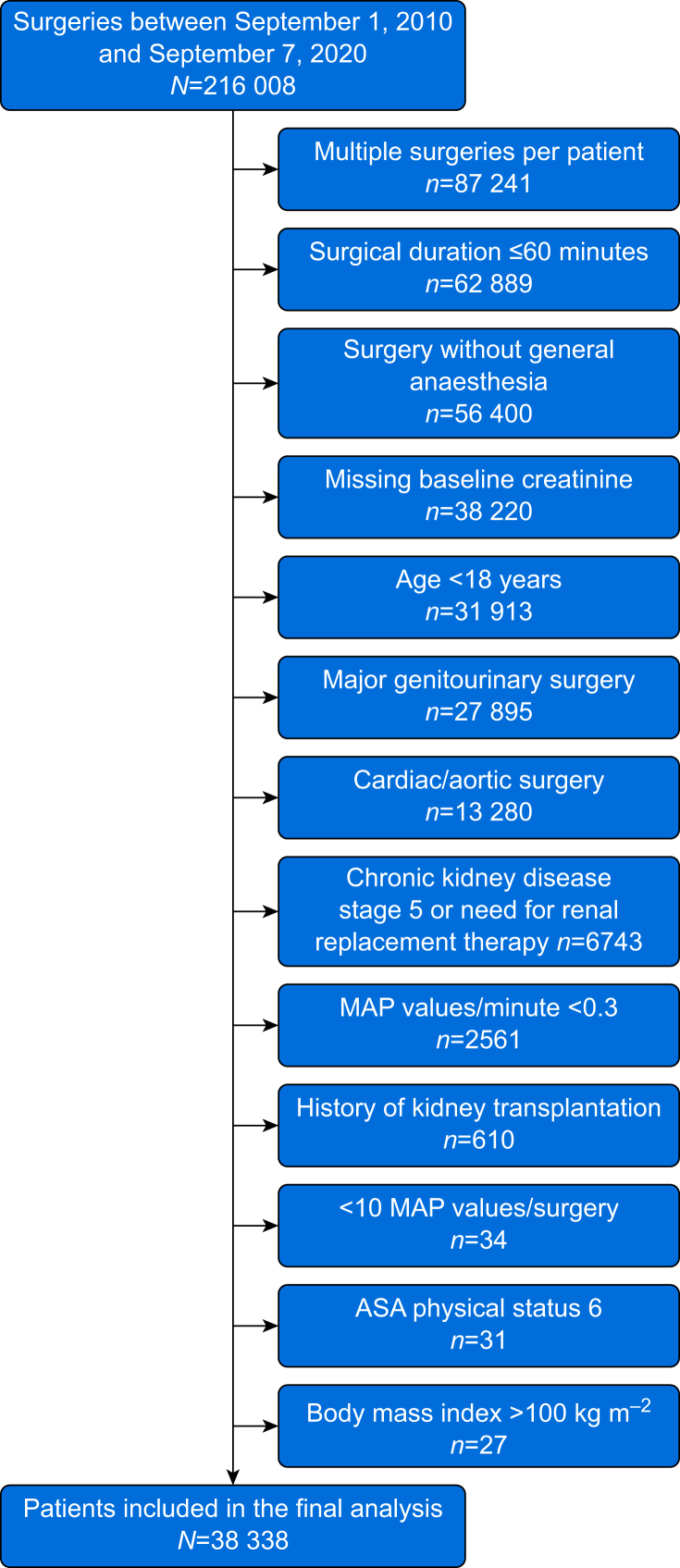
Flow chart illustrating study inclusion and exclusion criteria (one patient may have more than one reason for exclusion). ASA, American Society of Anesthesiologists; MAP, mean arterial pressure.

We included 38 338 patients with a mean [sd] age of 54.8 [18.6)] yr (Fig. 1, Table 1). Patients predominantly had general surgery (10 514 patients, 27.4%), trauma surgery (6177 patients, 16.1%), otolaryngology surgery (5853 patients, 15.3%), neurosurgery (5213 patients, 13.6%), orthopaedic surgery (3416 patients, 8.9%), and gynaecological surgery (1750 patients, 4.6%). The median (25th percentile, 75th percentile) duration of surgery was 132 (94, 194) min. The median total volume of crystalloid fluid administration was 760 (514, 1281) ml.

**Table 1:** Demographic, baseline, and clinical characteristics of patients. Data are presented as absolute number (percentage), mean (standard deviation), or median (25th, 75th percentile) for all patients, and for patients with acute kidney injury (AKI) and without AKI (non-AKI). ASA, American Society of Anesthesiologists.

Characteristic	All patients (N=38 338)	Non-AKI (*n*=36 881)	AKI (*n*=1457)	*P* value
Age (SD), yr	54.8 (18.6)	54.3 (18.6)	66.1 (15.5)	<0.001
Body mass index, kg m^−2^	26.3 (23.4, 30.0)	26.3 (23.4, 30.0)	26.6 (23.6, 30.9)	0.002
Male sex (%)	20 554 (53.6)	19 809 (53.7)	745 (51.1)	0.056
**Baseline risk factors**				
Baseline creatinine, mg dl^−1^	0.8 (0.7, 0.9)	0.8 (0.7, 0.9)	0.8 (0.6, 1.1)	0.004
Diabetes mellitus (%)	4021 (10.5)	3725 (10.1)	296 (20.3)	<0.001
Chronic arterial hypertension (%)	15 363 (40.1)	14 487 (39.3)	876 (60.1)	<0.001
Coronary artery disease or heart failure (%)	4968 (13.0)	4570 (12.4)	398 (27.3)	<0.001
**ASA physical status (%)**				<0.001
1	3579 (9.3)	3570 (9.7)	9 (0.6)	
2	22 625 (59.0)	22 237 (60.3)	388 (26.6)	
3	11 087 (28.9)	10 259 (27.8)	828 (56.8)	
4	998 (2.6)	783 (2.1)	215 (14.8)	
5	49 (0.1)	32 (0.1)	17 (1.2)	
**Clinical characteristics**				
Duration of surgery, min	132 (94, 194)	130 (93, 190)	204 (137, 324)	<0.001
Crystalloids, ml	760 (514, 1281)	746 (507, 1237)	1502 (899, 2431)	<0.001
Colloids, ml	0 (0, 0)	0 (0, 0)	0 (0, 500)	<0.001
Packed red blood cells, units	0 (0, 0)	0 (0, 0)	0 (0, 300)	<0.001
Fresh frozen plasma, units	0 (0, 0)	0 (0, 0)	0 (0, 0)	<0.001
Postoperative intensive care unit admission (%)	669 (1.7)	597 (1.6)	72 (4.9)	<0.001
**Type of surgery (%)**				<0.001
General	10 514 (27.4)	9704 (26.3)	810 (55.6)	
Trauma	6177 (16.1)	6025 (16.3)	152 (10.4)	
Otolaryngology	5853 (15.3)	5827 (15.8)	26 (1.8)	
Neurology	5213 (13.6)	5100 (13.8)	113 (7.8)	
Orthopaedic	3416 (8.9)	3326 (9.0)	90 (6.2)	
Oral and maxillofacial	2530 (6.6)	2514 (6.8)	16 (1.1)	
Gynaecology	1750 (4.6)	1692 (4.6)	58 (4.0)	
Peripheral vascular	1700 (4.4)	1560 (4.2)	140 (9.6)	
Eye	407 (1.1)	403 (1.1)	4 (0.3)	
Neuroradiology	226 (0.6)	210 (0.6)	16 (1.1)	
Dermatology	217 (0.6)	216 (0.6)	1 (0.1)	
Others	179 (0.5)	170 (0.5)	9 (0.6)	
Radiology	156 (0.4)	134 (0.4)	22 (1.5)	

Of the patients, 20 452 (53.3%) were given cafedrine/theodrenaline and 6028 (15.7%) were given norepinephrine; 4033 patients (10.5%) were given both cafedrine/theodrenaline and norepinephrine, 16 419 patients (42.8%) were given only cafedrine/theodrenaline, and 1995 patients (5.2%) were given only norepinephrine. Vasopressor therapy stratified by ASA physical status is shown in Supplementary Table S1.

**Fig 2:**
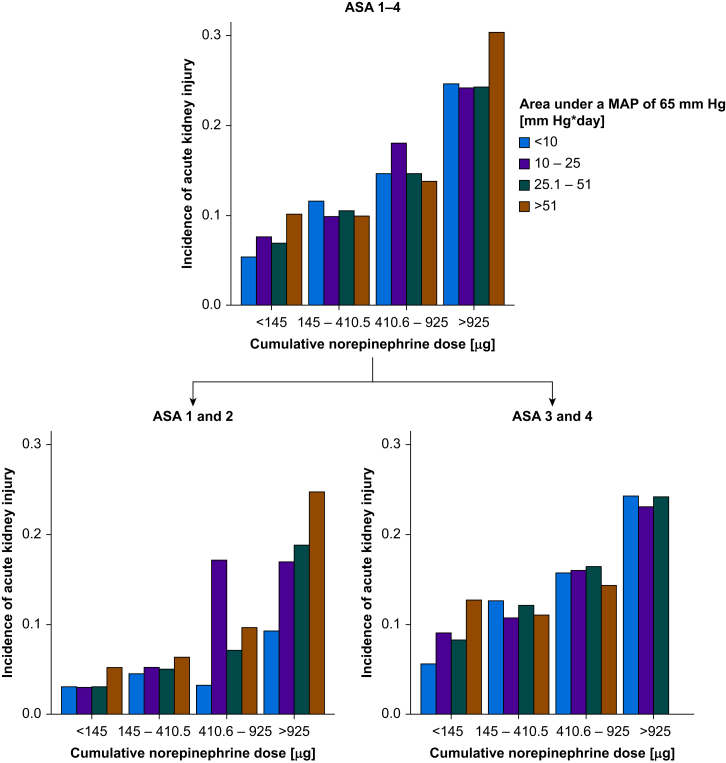
Bar charts illustrating the incidence of acute kidney injury (y axis) stratified by both quartiles of intraoperative cumulative norepinephrine dose (x axis) and quartiles of intraoperative hypotension (colour-coded) in all patients irrespective of their American Society of Anesthesiologists (ASA) physical status (upper chart), and separately in patients assigned ASA physical status 1 or 2 (lower left chart) *vs* 3 and 4 (lower right chart). The few patients assigned ASA physical status 5 were analysed together with those assigned ASA physical status 4. MAP, mean arterial pressure.

1457 patients (3.8%) developed AKI within the first 7 postoperative days, and 724 (2.0%) died during the hospital stay. Figure 2 shows the incidence of AKI stratified by both quartiles of intraoperative cumulative norepinephrine dose and quartiles of intraoperative hypotension.

The median cumulative duration patients had a MAP <65 mm Hg was 25 (8, 53) min in patients with AKI and 17 (4, 39) min in patients without AKI (*P*<0.001) (Table 2). The median area under a MAP of 65 mm Hg was 0.09 (0.02, 0.22) mm Hg∗day in patients with AKI and 0.05 (0.01, 0.14) mm Hg∗day in patients without AKI (*P*<0.001) (Table 2). The median cumulative intraoperative norepinephrine dose was 1.92 (range: 0.00–575.62) μg kg^−1^ in patients with AKI and 0.00 (range: 0.00–607.12) μg kg^−1^ in patients without AKI (*P*<0.001) (Table 2).

**Table 2:** Hypotension and vasopressors. Data are presented as absolute number (percentage) or median (25th, 75th percentile) for all patients, and for patients with acute kidney injury (AKI) and without AKI (non-AKI). MAP, mean arterial pressure.

Outcome	All patients(N=38 338)	Non-AKI(*n*=36 881)	AKI (*n*=1457)	*P* value
**Hypotension**				
Cumulative duration of MAP <65 mm Hg, min	17 (4, 40)	17 (4, 39)	25 (8, 53)	<0.001
Area under a MAP of 65 mm Hg, mm Hg∗day	0.05 (0.01, 0.15)	0.05 (0.01, 0.14)	0.09 (0.02, 0.22)	<0.001
Patients with a MAP <65 mm Hg (%)	32 235 (84.1)	30 903 (83.8)	1332 (91.4)	<0.001
**Vasopressors**				
Patients who received norepinephrine (%)	6028 (15.7)	5195 (14.1)	833 (57.2)	<0.001
Norepinephrine dose (μg kg^−1^)	0.00 (0.00, 0.00)	0.00 (0.00, 0.00)	1.92 (0.00, 13.09)	<0.001
Patients who received cafedrine/theodrenaline (%)	20 452 (53.3)	19 480 (52.8)	972 (66.7)	<0.001
Cafedrine/theodrenaline, ml	1.2 (0.6, 2.0)	1.2 (0.5, 2.0)	2.0 (1.0, 2.5)	<0.001
Patients who received only norepinephrine (%)	1995 (5.2)	1676 (4.5)	319 (21.9)	<0.001
Patients who received only cafedrine/theodrenaline (%)	16 419 (42.8)	15 961 (43.3)	458 (31.4)	<0.001
Patients who received norepinephrine and cafedrine/theodrenaline (%)	4033 (10.5)	3519 (9.5)	514 (35.3)	<0.001

The results of univariable logistic regression analyses are shown in Supplementary Table S2. In multivariable regression analysis including all patients, both the area under a MAP of 65 mm Hg (odds ratio 1.55 [95% CI 1.17–2.02] per mm Hg∗day; *P*=0.002) and the cumulative intraoperative norepinephrine dose (odds ratio 1.02 [95% CI 1.01–1.02] per μg kg^−1^; *P*<0.001) were independently associated with AKI, as were baseline patient risk factors including age, body mass index, diabetes mellitus, and procedural factors (including duration of surgery and total amounts of crystalloid and colloid fluids) (Table 3, Supplementary Table S3).

**Table 3:** Multivariable associations between exposures and acute kidney injury (all patients, N=38 338). Multivariable logistic regression analysis adjusting for the following covariables: cafedrine/theodrenaline, age, body mass index, sex, baseline creatinine, diabetes mellitus, chronic arterial hypertension, coronary artery disease or heart failure, American Society of Anesthesiologists physical status, duration of surgery, crystalloids, colloids, packed red blood cells, fresh frozen plasma, and type of surgery (Supplementary Table S3). MAP, mean arterial pressure.

Exposure	Odds ratio (95% CI)	*P* value
Area under a MAP of 65 mm Hg (mm Hg∗day)	1.55 (1.17, 2.02)	0.002
Norepinephrine dose (μg kg^−1^)	1.02 (1.01, 1.02)	<0.001

In multivariable regression analysis including only patients assigned ASA physical status 3 or 4, both the area under a MAP of 65 mm Hg (odds ratio 1.91 [95% CI 1.36–2.67] per mm Hg∗day; *P*<0.001) and the cumulative intraoperative norepinephrine dose (odds ratio 1.02 [95% CI 1.01–1.03] per μg kg^−1^; *P*<0.001) were independently associated with AKI (Table 4). In multivariable regression analysis including only patients assigned ASA physical status 1 or 2, the cumulative intraoperative norepinephrine dose was independently associated with AKI (odds ratio 1.02 [95% CI 1.00–1.03] per μg kg^−1^; *P*=0.006), but the area under a MAP of 65 mm Hg was not (odds ratio 1.00 [95% CI 0.56–1.66] per mm Hg∗day; *P*>0.9) (Table 4, Supplementary Table S4).

**Table 4:** Multivariable associations between exposures and acute kidney injury (divided in subgroups by American Society of Anesthesiologists physical status). Multivariable logistic regression analysis adjusting for the following covariables: cafedrine/theodrenaline, age, body mass index, sex, baseline creatinine, diabetes mellitus, chronic arterial hypertension, coronary artery disease or heart failure, Amercian Society of Anesthesiologists physical status, duration of surgery, crystalloids, colloids, packed red blood cells, fresh frozen plasma, and type of surgery (Supplementary Table S4). ASA, American Society of Anesthesiologists; MAP, mean arterial pressure.

Exposure	ASA physical status 1/2		ASA physical status 3/4	
	Odds ratio (95% CI)	*P* value	Odds ratio (95% CI)	*P* value
Area under a MAP of 65 mm Hg (mm Hg∗day)	1.00 (0.56, 1.66)	>0.9	1.91 (1.36, 2.67)	<0.001
Norepinephrine dose (μg kg^−1^)	1.02 (1.00, 1.03)	0.006	1.02 (1.01, 1.03)	<0.001

## Discussion

Our retrospective analysis of 38 338 adult male and female noncardiac surgery patients suggests that both intraoperative hypotension and the intraoperative cumulative norepinephrine dose are independently associated with postoperative AKI.

Consistent with previous observational research^5^, ^6^, ^7^^,^^14^^,^^23^ we found an association between intraoperative hypotension and AKI. Compared with patients who did not develop postoperative AKI, those who did had about one-third more intraoperative hypotension, as quantified by the area under a MAP of 65 mm Hg. In research, the area under a MAP threshold is an established marker of hypotension severity and duration,^21^ but it does not intuitively translate into measures clinically used to quantify hypotension. According to our results, the chance for developing postoperative AKI increased by more than 50% per 1 mm Hg∗day increase in the area under a MAP of 65 mm Hg. A 1 mm Hg∗day increase in the area under a MAP of 65 mm Hg translates into an increase of 24 mm Hg∗h and, for example, would result when the intraoperative MAP is 41 mm Hg for 1 h or 53 mm Hg for 2 h.

Interestingly, intraoperative hypotension was independently associated with postoperative AKI in our multivariable regression models including all patients and including only patients assigned ASA physical status 3 or 4, but not in the model including only patients assigned ASA physical status 1 or 2. This finding confirms a previous analysis suggesting that intraoperative hypotension is associated with postoperative AKI only in high-risk – but not in low-risk – patients.^14^

In general, baseline patient risk factors are more strongly associated with AKI than intraoperative hypotension.^7^^,^^14^ Our analysis, for example, suggests that the chance of developing postoperative AKI increases when patients are older or have diabetes mellitus. Most baseline patient risk factors are not modifiable before surgery. In contrast, intraoperative hypotension is amenable to interventions and can effectively be treated by clinicians,^24^ usually by giving vasopressors or fluids.

We found that the intraoperative cumulative norepinephrine dose was also independently associated with postoperative AKI in all three multivariable models (i.e. the model including all patients and the models including either only patients assigned ASA physical status 1 or 2 or only patients assigned ASA physical status 3 or 4). Considering all patients, the risk of developing postoperative AKI increased by 1.6% per 1 μg kg^−1^ increase in the cumulative intraoperative norepinephrine dose which would, for example, result when a patient is given norepinephrine at a rate of 0.05 μg kg^−1^ min^−1^ for 20 min. The risk of developing postoperative AKI would thus increase by 16% if norepinephrine was given at 0.076 μg kg^−1^ min^−1^ for 132 min (the median duration of surgery in our study).

Most previous analyses on the relationship between intraoperative hypotension and postoperative AKI did not consider intraoperative vasopressor requirements as a potential independent risk factor for AKI.^4^^,^^5^^,^^7^^,^^14^^,^^25^ A retrospective cohort study of 830 elective noncardiac surgery patients who were at least 65 yr of age reported an association between the cumulative intraoperative vasopressor dose and postoperative AKI.^11^ Specifically, the cumulative intraoperative dose of any vasopressor >20 mg was independently associated with AKI (odds ratio 2.47 [95% CI 1.34–4.58]; *P*=0.004), with every 5 mg increase in the cumulative intraoperative vasopressor dose increasing the AKI risk by 11% (95% CI 3–19%; *P*=0.06).^11^ Similarly, a retrospective analysis of 32 250 patients who had major abdominal surgery suggests that increasing the vasopressor dose from 0 to 0.04 μg kg^−1^ min^−1^ of norepinephrine equivalents would increase the risk of AKI 1.8-fold.^10^ In an international observational study of more than 10 000 major surgery patients requiring postoperative treatment in an intensive care or high-dependency unit, the use of vasopressors was associated with an increased risk of developing postoperative AKI.^12^

In patients assigned ASA physical status 1 or 2 (but not 3 and 4), the cumulative intraoperative dose of cafedrine/theodrenaline was associated with postoperative AKI. One may speculate that clinicians use cafedrine/theodrenaline in low-risk patients and norepinephrine in high-risk patients who are more likely to develop profound intraoperative hypotension. More observational studies and robust interventional trials are needed to investigate the relationship between different vasopressors and postoperative AKI. A large multicentre pilot trial of 3626 adults having noncardiac surgery showed that such trials are warranted and feasible.^26^

The optimal haemodynamic treatment strategy to avoid AKI in patients having surgery remains unknown. During the past decade, clinicians seem to increasingly focus on avoiding profound hypotension, often by the liberal use of vasopressors. A retrospective analysis of fluid and vasopressor therapy in 26 US hospitals reported that between 2015 and 2019 major abdominal surgery patients were given less fluids but more vasopressors.^10^ Although the duration of hypotension decreased during the study period, the incidence of postoperative AKI slightly increased (from 11.4% in 2016 to 13% in 2019).^10^

Although our retrospective observational analysis suggests that both intraoperative hypotension and the intraoperative cumulative norepinephrine dose were independently associated with postoperative AKI, it does not allow a formal analysis of a causal relationship between intraoperative hypotension, the administration of vasopressors, and AKI. Norepinephrine, cafedrine/theodrenaline, and intravenous fluids (crystalloids and colloids) have a direct effect on blood pressure and are given to prevent or treat hypotension, and may be linked to the development of AKI. We thus treated norepinephrine, cafedrine/theodrenaline, and intravenous fluids (crystalloids and colloids) as mediators in our analyses. Although we adjusted our regression models for known risk factors for AKI and potential clinical confounders, our findings may be influenced by residual confounding.

That there was no standardised treatment protocol for intraoperative hypotension may reflect routine care in most institutions. However, clinicians' personal preferences likely influenced decisions regarding fluid and vasopressor therapy. Finally, we excluded patients in whom preoperative baseline serum creatinine values were missing. We cannot quantify whether and how these patients differed from patients included in the analysis. However, it is likely that patients in whom serum creatinine was not measured before surgery were relatively young and healthy and had minor surgery.

### Conclusions

Both intraoperative hypotension and the intraoperative cumulative norepinephrine dose were independently associated with postoperative acute kidney injury in female and male adults having noncardiac surgery. Pending results of trials testing whether these relationships are causal, it seems prudent to avoid both profound hypotension and high norepinephrine doses.

## Authors’ contributions

Study conception/design: BS, MS, CKa, MM

Data analysis/interpretation: all authors

Statistical analysis: CKa, KR

Drafting of manuscript: BS, MS, KK, KR, MH

Critical revision of article for important intellectual content: all authors

Final approval of the version to be published: all authors

Agreement to be accountable for all aspects of the work, thereby ensuring that questions related to the accuracy or integrity of any part of the work are appropriately investigated and resolved: all authors

MS, CKa, KR, and MH had full access to all of the data in the study and are responsible for the integrity of the data and the accuracy of the data analysis

## Funding

Supported solely from institutional and or departmental sources.

## Declarations of interest

BS is a consultant for and has received institutional restricted research grants and honoraria for giving lectures from Edwards Lifesciences (Irvine, CA, USA). BS is a consultant for Philips North America (Cambridge, MA, USA) and has received honoraria for giving lectures from Philips Medizin Systeme Böblingen (Böblingen, Germany). BS has received institutional restricted research grants and honoraria for giving lectures from Baxter (Deerfield, IL, USA). BS is a consultant for and has received institutional restricted research grants and honoraria for giving lectures from GE Healthcare (Chicago, IL, USA). BS has received institutional restricted research grants and honoraria for giving lectures from CNSystems Medizintechnik (Graz, Austria). BS is a consultant for Maquet Critical Care (Solna, Sweden). BS has received honoraria for giving lectures from Getinge (Gothenburg, Sweden). BS is a consultant for and has received institutional restricted research grants and honoraria for giving lectures from Pulsion Medical Systems (Feldkirchen, Germany). BS is a consultant for and has received institutional restricted research grants and honoraria for giving lectures from Vygon (Aachen, Germany). BS is a consultant for and has received institutional restricted research grants from Retia Medical (Valhalla, NY, USA). BS has received honoraria for giving lectures from Masimo (Neuchâtel, Switzerland). BS is a consultant for Dynocardia (Cambridge, MA, USA). BS has received institutional restricted research grants from Osypka Medical (Berlin, Germany). BS received honoraria for giving lectures from Ratiopharm (Ulm, Germany). BS was a consultant for and has received institutional restricted research grants from Tensys Medical (San Diego, CA, USA). BS is an Editor of the British Journal of Anaesthesia. MS is a consultant for Edwards Lifesciences (Irvine, CA, USA) and has received institutional research funding for investigator-initiated trials and honoraria for giving lectures from Edwards Lifesciences, has received honoraria for giving lectures from AMOMED (Vienna, Austria), and has received honoraria for giving lectures from Orion Pharma (Hamburg, Germany). MS has received honoraria for giving lectures from Philips Medizin Systeme Böblingen (Böblingen, Germany). ES received honoraria for giving lectures from Edwards Lifesciences (Irvine, CA, USA) and Orion Pharma (Hamburg, Germany). MF is a consultant for Edwards Lifesciences (Irvine, CA, USA) and has received honoraria for consulting and giving lectures from CNSystems Medizintechnik (Graz, Austria). KK is a consultant for and has received honoraria for giving lectures from Edwards Lifesciences (Irvine, CA, USA). KK is a consultant for Vygon (Aachen, Germany). FB reports grants from German Federal Ministry of Education and Research, grants from German Federal Ministry of Health, grants from Berlin Institute of Health, personal fees from Elsevier Publishing, grants from Hans Böckler Foundation, other from Robert Koch Institute, grants from Einstein Foundation, grants from Berlin University Alliance, personal fees from Medtronic, personal fees from GE Healthcare, grants from German Research Foundation, and grants from Federal Joint Committee, outside the submitted work. MH has received honoraria for consulting and giving lectures from Edwards Lifesciences (Irvine, CA, USA) and Baxter (Deerfield, IL, USA). CKa, CH, CKo, DL, MM, and KR have no conflicts of interest to declare.
