# Corrigendum to ‘Association of intraoperative hypotension and cumulative norepinephrine dose with postoperative acute kidney injury in patients having noncardiac surgery: a retrospective cohort analysis’ [*Br J Anaesth* 2025; 134: 54–62]

**DOI:** 10.1016/j.bja.2025.03.004

**Published:** 2025-04-05

**Authors:** Bernd Saugel, Michael Sander, Christian Katzer, Christian Hahn, Christian Koch, Dominik Leicht, Melanie Markmann, Emmanuel Schneck, Moritz Flick, Karim Kouz, Kerstin Rubarth, Felix Balzer, Marit Habicher

**Affiliations:** 1 Department of Anesthesiology, Center of Anesthesiology and Intensive Care Medicine, University Medical Center Hamburg-Eppendorf, Hamburg, Germany; 2 Outcomes Research Consortium, Cleveland, OH, USA; 3 Department of Anaesthesiology, Intensive Care Medicine and Pain Medicine, University Hospital Giessen, Justus-Liebig University Giessen, Giessen, Germany; 4 Institute of Medical Informatics, Charité–Universitätsmedizin Berlin, Corporate Member of Freie Universität Berlin and Humboldt-Universität zu Berlin, Berlin, Germany; 5 Institute of Biometry and Clinical Epidemiology, Charité–Universitätsmedizin Berlin, Corporate Member of Freie Universität Berlin and Humboldt-Universität zu Berlin, Berlin, Germany

The authors regret that Figure 2 was incorrect. The correct figure appears below. The authors would like to apologise for any inconvenience caused.

**Fig. 2:**
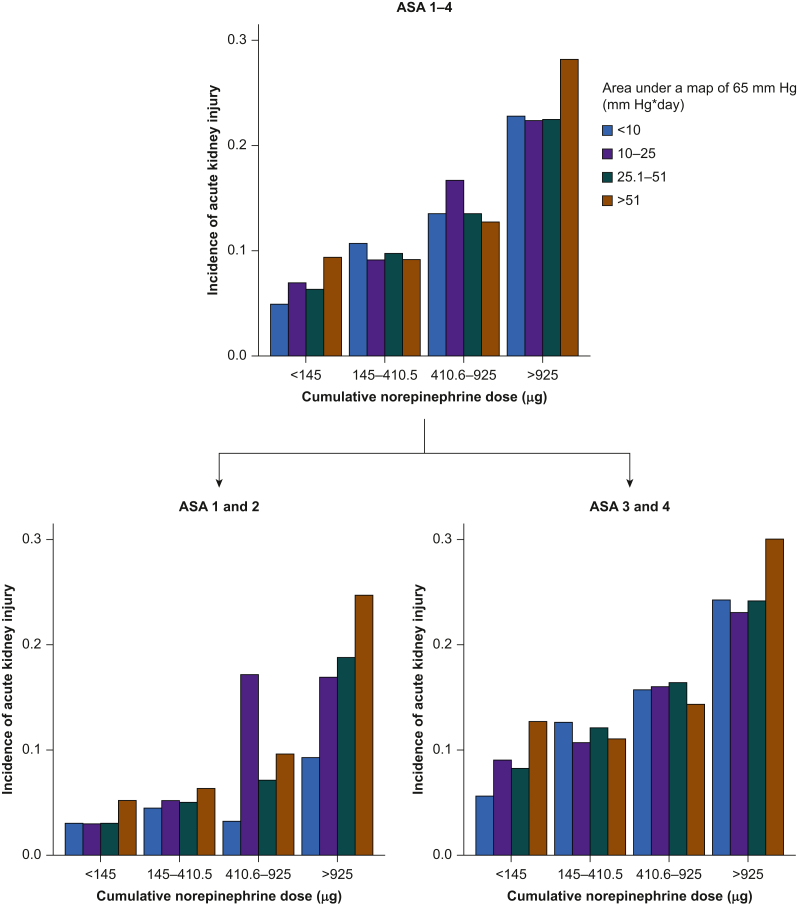
Bar charts illustrating the incidence of acute kidney injury (y axis) stratified by both quartiles of intraoperative cumulative norepinephrine dose (x axis) and quartiles of intraoperative hypotension (colour-coded) in all patients irrespective of their ASA physical status (upper chart) and separately in patients assigned ASA physical status 1 or 2 (lower left chart) *vs* 3 and 4 (lower right chart). The few patients assigned ASA physical status 5 were analysed together with those assigned ASA physical status 4. ASA, American Society of Anesthesiologists; MAP, mean arterial pressure.

