# Supplementary material for: Association of intraoperative hypotension and cumulative norepinephrine dose with postoperative acute kidney injury in patients having noncardiac surgery: a retrospective cohort analysis
Source: Br J Anaesth. 2024 Dec 12;134(1):54–62. doi: 10.1016/j.bja.2024.11.005 (PMC11718363; doi:10.1016/j.bja.2024.11.005)
Supplement: Multimedia component 2 [file mmc2.pdf]

Supplementary Figure 2: Directed acyclic graph illustrating the relationships between variables (exposure: norepinephrine dose).

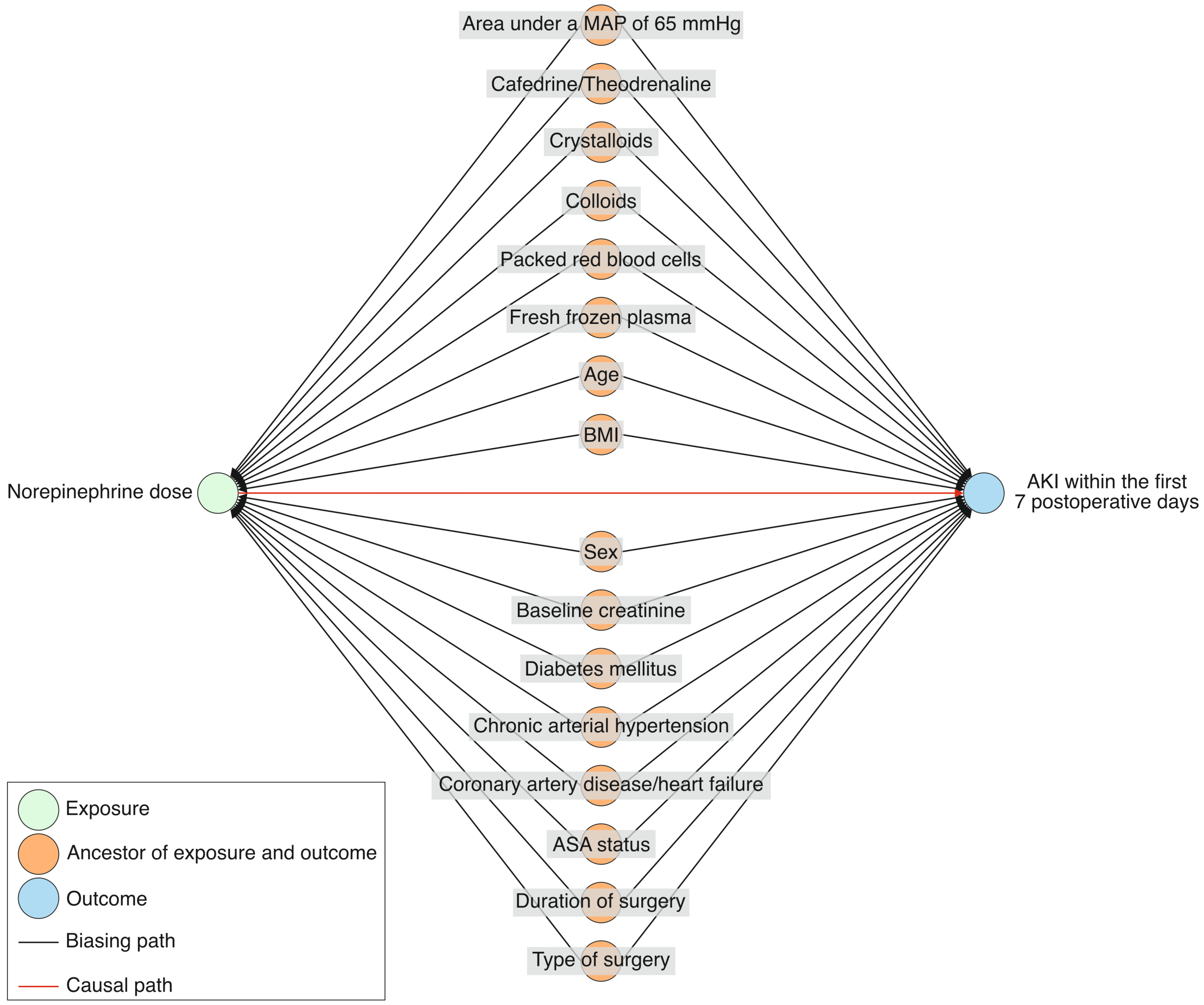

AKI, acute kidney injury; ASA: American Society of Anesthesiologists; BMI, body mass index; MAP, mean arterial pressure; Norepinephrine dose: cumulative intraoperative norepinephrine dose.
