# Supplementary material for: Association of intraoperative hypotension and cumulative norepinephrine dose with postoperative acute kidney injury in patients having noncardiac surgery: a retrospective cohort analysis
Source: Br J Anaesth. 2024 Dec 12;134(1):54–62. doi: 10.1016/j.bja.2024.11.005 (PMC11718363; doi:10.1016/j.bja.2024.11.005)
Supplement: Multimedia component 3 [file mmc3.pdf]

Supplementary Table 1: Vasopressor therapy stratified by American Society of Anesthesiologists physical status class

Vasopressor therapy	All patients (N = 38,338)	ASA I (n = 3,579)	ASA II (n = 22,625)	ASA III (n = 11,087)	ASA IV+V (n = 1,047)	p value
Patients who received norepinephrine (%)	6,028 (15.7)	48 (1.3)	1,714 (7.6)	3,506 (31,6)	760 (72.6)	<0.001
Patients who received cafedrine/theodrenaline (%)	20,452 (53.3)	939 (26.2)	11,435 (50.5)	7,624 (68.8)	454 (43.4)	<0.001
Patients who only received norepinephrine (%)	1,995 (5.2)	14 (0.4)	362 (1.6)	1,140 (10.3)	479 (45.7)	<0.001
Patients who only received cafedrine/theodrenaline (%)	16,419 (42.8)	905 (25.3)	10,083 (44.6)	5,258 (47.4)	173 (16.5)	<0.001
Patients who received norepinephrine and cafedrine/theodrenaline (%)	4,033 (10.5)	34 (0.9)	1,352 (6.0)	2,366 (21.3)	281 (26.8)	<0.001

ASA, American Society of Anesthesiologists.
