# Supplementary material for: Association of intraoperative hypotension and cumulative norepinephrine dose with postoperative acute kidney injury in patients having noncardiac surgery: a retrospective cohort analysis
Source: Br J Anaesth. 2024 Dec 12;134(1):54–62. doi: 10.1016/j.bja.2024.11.005 (PMC11718363; doi:10.1016/j.bja.2024.11.005)
Supplement: Multimedia component 4 [file mmc4.pdf]

Supplementary Table 2: Univariable associations between exposures and acute kidney injury (all patients, N = 38,338)

Exposure	Odds Ratio (95% CI)	p value
Area under a MAP of 65 mmHg (mmHg x day)	2.78 (2.35, 3.29)	<0.001
Norepinephrine dose (µg/kg)	1.05 (10.5, 1.05)	<0.001
Cafedrine/theodrenaline (2 ml)	1.39 (1.27, 1.52)	<0.001
Age (year)	1.04 (1.04, 1.04)	<0.001
Body mass index >30 kg/m^2 (yes vs. no)	1.02 (1.02, 1.03)	<0.001
Sex (female vs. male)	1.11 (1.00, 1.23)	0.053
Baseline creatinine (mg/dl)	1.92 (1.78, 2.08)	<0.001
Diabetes mellitus (yes vs. no)	2.27 (1.99, 2.59)	<0.001
Chronic arterial hypertension (yes vs. no)	2.33 (2.09, 2.59)	<0.001
Coronary artery disease/heart failure (yes vs. no)	2.66 (2.36, 2.99)	<0.001
**ASA physical status class (reference: I)**		
II	6.92 (3.57, 13.41)	<0.001
III	32.02 (16.58, 61.81)	<0.001
IV	112.92 (57.77, 220.69)	<0.001
Duration of surgery (min)	1.01 (1.01, 1.01)	<0.001
Crystalloids (500 ml)	1.35 (1.33, 1.38)	<0.001
Colloids (500 ml)	2.37 (2.22, 2.53)	<0.001
Packed red blood cells (units)	1.67 (1.60, 1.73)	<0.001
Fresh frozen plasma (units)	1.65 (1.58, 1.74)	<0.001
**Type of surgery (reference: orthopaedic)**		
General	3.09 (2.47, 3.85)	<0.001
Trauma	0.93 (0.72, 1.21)	0.603
Otolaryngologic	0.17 (0.11, 0.26)	<0.001
Neurology	0.82 (0.62, 1.08)	0.162
Oral and maxillofacial	0.24 (0.14, 0.40)	<0.001
Gynaecology	1.27 (0.91, 1.77)	0.167
Peripheral vascular	3.32 (2.53, 4.35)	<0.001
Eye	0.37 (0.13, 1.00)	0.051
Neuroradiology	2.82 (1.63, 4.88)	<0.001
Dermatology	0.17 (0.02, 1.23)	0.080
Others	1.96 (0.97, 3.95)	0.061
Radiology	6.07 (3.69, 9.98)	<0.001

ASA, American Society of Anesthesiologists; MAP, mean arterial pressure.
