# Supplementary material for: Association of intraoperative hypotension and cumulative norepinephrine dose with postoperative acute kidney injury in patients having noncardiac surgery: a retrospective cohort analysis
Source: Br J Anaesth. 2024 Dec 12;134(1):54–62. doi: 10.1016/j.bja.2024.11.005 (PMC11718363; doi:10.1016/j.bja.2024.11.005)
Supplement: Multimedia component 5 [file mmc5.pdf]

Supplementary Table 3: Multivariable associations between exposures and acute kidney injury (all patients, N = 38,338)

Exposure	Odds Ratio (95% CI)	p value
Area under a MAP of 65 mmHg (mmHg x day)	1.55 (1.17, 2.02)	0.002
Norepinephrine dose (µg/kg)	1.02 (1.01 1.02)	<0.001
Cafedrine/theodrenaline (2 ml)	1.03 (0.98, 1.07)	0.2
Age (year)	1.02 (1.02, 1.03)	<0.001
Body mass index >30 kg/m^2 (yes vs. no)	1.01 (1.00, 1.03)	0.006
Sex (female vs. male)	1.21 (1.05, 1.40)	0.008
Baseline creatinine (mg/dl)	1.44 (1.30, 1.60)	<0.001
Diabetes mellitus (yes vs. no)	1.21 (1.02, 1.45)	0.032
Chronic arterial hypertension (yes vs. no)	1.06 (0.90, 1.25)	0.5
Coronary artery disease/heart failure (yes vs. no)	0.92 (0.77, 1.10)	0.4
**ASA physical status class (reference: I)**		
II	2.05 (1.06, 4.65)	0.054
III	3.81 (1.94, 8.66)	<0.001
IV	9.39 (4.54, 22.13)	<0.001
Duration of surgery (min)	1.00 (1.00, 1.00)	<0.001
Crystalloids (500 ml)	1.06 (1.01, 1.11)	0.016
Colloids (500 ml)	1.35 (1.22, 1.50)	<0.001
Packed red blood cells (units)	1.12 (1.03, 1.21)	0.010
Fresh frozen plasma (units)	0.86 (0.78, 0.95)	0.002
**Type of surgery (reference: orthopaedic)**		
General	1.67 (1.30, 2.18)	<0.001
Trauma	1.25 (0.93, 1.70)	0.14
Otolaryngologic	0.27 (0.16, 0.45)	<0.001
Neurology	0.42 (0.30, 0.60)	<0.001
Oral and maxillofacial	0.11 (0.04, 0.22)	<0.001
Gynaecology	1.51 (0.97, 2.31)	0.065
Peripheral vascular	1.11 (0.79, 1.57)	0.6
Eye	0.45 (0.11, 1.23)	0.2
Neuroradiology	1.33 (0.60, 2.66)	0.4
Dermatology	0.32 (0.02, 1.47)	0.3
Others	1.91 (0.82, 3.98)	0.1
Radiology	2.75 (1.23, 5.50)	0.007

ASA, American Society of Anesthesiologists; MAP, mean arterial pressure.
