# Supplementary material for: Association of intraoperative hypotension and cumulative norepinephrine dose with postoperative acute kidney injury in patients having noncardiac surgery: a retrospective cohort analysis
Source: Br J Anaesth. 2024 Dec 12;134(1):54–62. doi: 10.1016/j.bja.2024.11.005 (PMC11718363; doi:10.1016/j.bja.2024.11.005)
Supplement: Multimedia component 6 [file mmc6.pdf]

Supplementary Table 4: Multivariable associations between exposures and acute kidney injury (divided in subgroups by ASA physical status class)

	ASA physical status class I/II		ASA physical status class III/IV	
**Exposure**	**Odds Ratio (95% CI)**	**p value**	**Odds Ratio (95% CI)**	**p value**
Area under a MAP of 65 mmHg (mmHg x day)	1.00 (0.56, 1.66)	>0.9	1.91 (1.36, 2.67)	<0.001
Norepinephrine dose (µg/kg)	1.02 (1.00, 1.03)	0.006	1.02 (1.01, 1.03)	<0.001
Cafedrine/theodrenaline (2 ml)	1.20 (1.01, 1.42)	0.030	1.02 (0.94, 1.06)	0.5
Age (year)	1.03 (1.02, 1.04)	<0.001	1.02 (1.01, 1.02)	<0.001
Body mass index >30 kg/m^2 (yes vs. no)	1.03 (1.00, 1.05)	0.051	1.01 (1.00, 1.02)	0.13
Sex (female vs. male)	1.14 (0.88, 1.47)	0.3	1.25 (1.05, 1.49)	0.013
Baseline creatinine (mg/dl)	1.32 (0.90, 1.79)	0.1	1.44 (1.29, 1.61)	<0.001
Diabetes mellitus (yes vs. no)	1.64 (1.15, 2.30)	0.005	1.11 (0.90, 1.35)	0.3
Chronic arterial hypertension (yes vs. no)	1.17 (0.89, 1.54)	0.3	0.97 (0.79, 1.19)	0.7
Coronary artery disease/heart failure (yes vs. no)	0.85 (0.51, 1.35)	0.5	0.97 (0.80, 1.17)	0.8
ASA physical status class II (reference: I)	1.78 (0.89, 4.14)	0.14		
ASA physical status class IV (reference: III)			2.38 (1.79, 3.14)	<0.001
Duration of surgery (min)	1.00 (1.00, 1.01)	<0.001	1.00 (1.00, 1.00)	<0.001
Crystalloids (500 ml)	1.08 (1.00, 1.17)	0.061	1.03 (0.97, 1.10)	0.3
Colloids (500 ml)	1.27 (1.07, 1.51)	0.007	1.33 (1.17, 1.52)	<0.001
Packed red blood cells (units)	1.10 (0.93, 1.30)	0.2	1.14 (1.03, 1.26)	0.010
Fresh frozen plasma (units)	0.86 (0.73, 1.02)	0.089	0.87 (0.77, 0.97)	0.014
**Type of surgery (reference: orthopaedic)**				
General	1.49 (1.03, 2.20)	0.039	1.81 (1.28, 2.64)	0.001
Trauma	1.03 (0.64, 1.65)	0.9	1.46 (0.98, 2.21)	0.071

Otolaryngologic	0.16 (0.06, 0.38)	<0.001	0.39 (0.20, 0.73)	0.005
Neurology	0.31 (0.18, 0.54)	<0.001	0.52 (0.33, 0.81)	0.004
Oral and maxillofacial	0.12 (0.03, 0.35)	<0.001	0.11 (0.03, 0.30)	<0.001
Gynaecology	2.06 (1.15, 3.64)	0.014	0.97 (0.46, 1.91)	>0.9
Peripheral vascular	0.95 (0.37, 2.08)	0.9	1.20 (0.79, 1.86)	0.4
Eye	0.55 (0.03, 2.58)	0.6	0.40 (0.07, 1.39)	0.2
Neuroradiology	2.52 (0.39, 8.96)	0.2	1.19 (0.46, 2.65)	0.7
Dermatology	0.00 (0.00, 0.00)	>0.9	0.76 (0.04, 3.75)	0.8
Others	0.00 (0.00, 0.00)	>0.9	1.80 (0.70, 4.10)	0.2
Radiology	2.05 (0.11, 10.44)	0.5	2.91 (1.22, 6.37)	0.010

ASA, American Society of Anesthesiologists; MAP, mean arterial pressure.
